# P-2170. CMV T-cell Immunity Panel (TCIP)-Guided Secondary CMV Prophylaxis Duration in Lung Transplant Recipients

**DOI:** 10.1093/ofid/ofae631.2324

**Published:** 2025-01-29

**Authors:** Andres E Franceschi Coll, Marco Aurelio Diaz, Hassan Alshaker, Jawad Safiia, Dimitrios G Moshovitis, Christine Atallah, Paul Sakr, Dimitrios Farmakiotis, Sophia Koo

**Affiliations:** Brigham and Women's Hospital, Boston, Massachusetts; Brigham and Women's Hospital, Boston, Massachusetts; Brigham and Women's Hospital, Boston, Massachusetts; Brigham and Women's Hospital, Boston, Massachusetts; Brigham and Women's Hospital, Boston, Massachusetts; Brigham and Women's Hospital, Boston, Massachusetts; Brigham and Women's Hospital, Boston, Massachusetts; Division of Infectious Diseases, The Warren Alpert Medical School of Brown University, providence, Rhode Island; Brigham and Women's Hospital, Dana-Farber Cancer Institute, Boston, MA

## Abstract

**Background:**

CMV infection is a complication in lung transplant recipients (LTR), with associated morbimortality. There are many antiviral regimens, but extended use can lead to toxicity. The use of a CMV-specific TCIP (CMV inSIGHT, Viracor Eurofins) is being evaluated for CMV risk stratification in solid organ transplantation. In LTR with prior CMV reactivation or infection, it is challenging to determine the duration of secondary prophylaxis using clinical parameters alone. We aimed to assess if TCIP results can guide clinicians on the duration of secondary CMV prophylaxis in LTR.

**Table 1:**
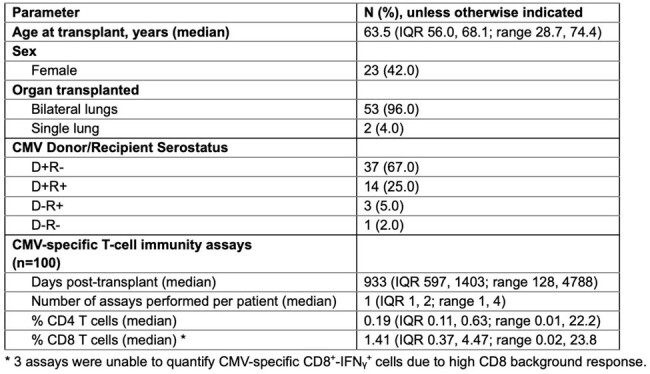
Baseline Characteristics.

**Methods:**

A retrospective cohort study in an 826-bed hospital in 55 adults (≥18 years) LTR from 2010-2024 and underwent TCIP to guide the duration of secondary prophylaxis after a prior CMV episode post-transplant (**Table 1**). We assessed CMV antivirals, TCIP results, post-TCIP treatment decisions, and post-TCIP reactivations. We considered decision-making concordant with TCIP results (TCIP-C) if antivirals were stopped in response to both CD4+ and CD8+ CMV-specific T-cells > 0.20% (a positive result) or continued prophylaxis if both were ≤ 0.20%. We considered decision-making discordant (TCIP-D) if antivirals were continued despite both values > 0.20 or discontinued despite both being ≤ 0.20%.

**Table 2:**
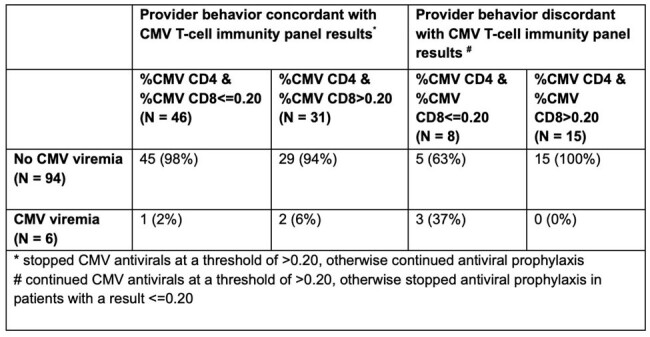
IGRA results with either CD4 or CD8 CMV-specific T-cell subpopulations as a threshold.

**Results:**

100 unique TCIP were performed, with TCIP-C in 77 and TCIP-D in 23 (**Table 2**). In 31 TCIP-positive patients whose antivirals were discontinued, 29 (94%) had no further CMV viremia. In 8 patients with negative TCIP in whom prophylaxis was stopped (mostly due to patient preference), 3 (37%) developed viremia, and in 15 patients with a positive test who continued therapy, 100% had no further viremia. Of the 6 reactivations (1 CMV syndrome in a patient with a positive TCIP, 5 asymptomatic viremia), 4 occurred in patients with a negative TCIP; 3 in patients who stopped antivirals despite impaired immunity. Reactivation risk was higher in TCIP-negative patients discontinuing prophylaxis compared to TCIP-positive patients stopping antivirals (Fisher’s exact p=0.049) or TCIP-negative patients on prophylaxis (p=0.008).

**Conclusion:**

CMV TCIP appears to be a useful adjunct in the management of secondary CMV antiviral prophylaxis in LTR. Prophylaxis can be safely discontinued in most LTR with a positive TCIP result.

**Disclosures:**

Dimitrios Farmakiotis, M.D., Astra Zeneca: Grant/Research Support|Viracor: Advisor/Consultant|Viracor: Honoraria

